# Malaria associated symptoms in pregnant women followed-up in Benin

**DOI:** 10.1186/1475-2875-10-72

**Published:** 2011-03-31

**Authors:** Bich-Tram Huynh, Nadine Fievet, Gildas Gbaguidi, Sophie Borgella, Blaise Guézo Mévo, Achille Massougbodji, Philippe Deloron, Michel Cot

**Affiliations:** 1 IRD/UMR 216 - Mère et enfant face aux infections tropicales, Faculté des sciences pharmaceutiques - 4 avenue de l'Observatoire, 75270 Paris cedex 06, France; 2 Université Paris Descartes, Faculté des sciences pharmaceutiques - 4 avenue de l'Observatoire, 75270 Paris cedex 06, France; 3 Hôpital de Comé, BP 294 Comé, Bénin; 4 Faculté des Sciences de la Santé (FSS), UER de Parasitologie, Université de Cotonou 01BP188, Cotonou, Bénin

## Abstract

**Background:**

It is generally agreed that in high transmission areas, pregnant women have acquired a partial immunity to malaria and when infected they present few or no symptoms. However, longitudinal cohort studies investigating the clinical presentation of malaria infection in pregnant women in stable endemic areas are lacking, and the few studies exploring this issue are unconclusive.

**Methods:**

A prospective cohort of women followed monthly during pregnancy was conducted in three rural dispensaries in Benin from August 2008 to September 2010. The presence of symptoms suggestive of malaria infection in 982 women during antenatal visits (ANV), unscheduled visits and delivery were analysed. A multivariate logistic regression was used to determine the association between symptoms and a positive thick blood smear (TBS).

**Results:**

During routine ANVs, headache was the only symptom associated with a higher risk of positive TBS (aOR = 1.9; p < 0.001). On the occasion of unscheduled visits, fever (aOR = 5.2; p < 0.001), headache (aOR = 2.1; p = 0.004) and shivering (aOR = 3.1; p < 0.001) were significantly associated with a malaria infection and almost 90% of infected women presented at least one of these symptoms. Two thirds of symptomatic malaria infections during unscheduled visits occurred in late pregnancy and long after the last intermittent preventive treatment dose (IPTp).

**Conclusion:**

The majority of pregnant women were symptomless during routine visits when infected with malaria in an endemic stable area. The only suggestive sign of malaria (fever) was associated with malaria only on the occasion of unscheduled visits. The prevention of malaria in pregnancy could be improved by reassessing the design of IPTp, i.e. by determining an optimal number of doses and time of administration of anti-malarial drugs.

## Background

In sub-Saharan Africa, 30 million women become pregnant each year[1]. Consequences of malaria in pregnancy (MiP) are a threat both to these mothers and to their newborns. MiP increases the risk of maternal anaemia, and of low birth weight (LBW) [2,3]. LBW is the single most important determinant of mortality during the first year of life in African infants [4]. It has been estimated that between 75,000 and 200,000 newborn deaths occur each year as a direct result of LBW due to malaria in pregnancy [1,4,5].

To prevent the consequences of MiP, the WHO recommends an intermittent preventive treatment during pregnancy (IPTp), an adequate management of clinical malaria, and the use of insecticide-treated nets. IPTp consists in the administration of two curative doses of sulphadoxine/pyrimethamine (SP), at least four weeks apart and beginning at the quickening[1], regardless to the women's malaria status. It is generally agreed that in high transmission areas, pregnant women have acquired a partial immunity to the disease and when infected they present no or few symptoms[6-8]. Therefore, even if the women remain asymptomatic and do not get a curative treatment or do not attend frequently antenatal visits (ANV), it is likely that SP IPTp has a long acting prophylactic effect which protects them for the duration of the pregnancy [9].

Longitudinal cohort studies investigating the clinical presentation of malaria infection in pregnant women in stable endemic areas are lacking, and only a limited number of studies have focused on this issue[10-12]. Consequently, there is a need to ascertain throughout pregnancy whether in malaria highly endemic areas, infected pregnant women are really asymptomatic or not, and if there are symptoms, to evaluate their actual incidence and intensity.

The STOPPAM project (Stategy TO Prevent Pregnancy Associated Malaria) consists in a prospective cohort of 1000 pregnant women in Benin. This longitudinal follow-up offered us the unique opportunity to assess the clinical presentation of infected women during the whole duration of pregnancy.

## Methods

The STOPPAM project has been conceived to study the underlying immunopathological processes causing poor outcomes in MiP, through the characterization and measurements of antibody and cellular immunological responses during pregnancy, at delivery and during the infant's first year of life.

In this study, 1,000 pregnant women were followed-up in each of two sites: Benin and Tanzania. Specifically in Benin, 200 newborns to these mothers were followed-up during their first year of life to investigate the immunological responses in the infants.

### Study area, population

The study took place in the Mono province, located 70 km west of the economical capital of Benin, Cotonou. It is a high transmission area, with two peaks during the rainy seasons: from April to June, and from September to November. The entomological inoculation rate is 35-60 infective bites per person and per year [13]. Three dispensaries were involved: Come, Akodeha and Ouedeme Pedah, 10 km away from each other. Come is a semi-rural area and the two other health centres are located in a more rural surrounding. The principal occupations of the inhabitants are farming, fishing and trading.

There are one district hospital (which is the referral hospital for the study), three government-run health dispensaries and 11 private clinics in the study area. Five nurses have been recruited and trained as "project assistants" to fill questionnaires and to collect blood samples from the study participants. Local midwives, nurses in charge of the health centre and the project assistants worked in close collaboration.

### Study design

Pregnant women meeting the following criteria: gestational age under 24 weeks, living for more than six months within 15 km from the dispensary and having planned to deliver at the hospital, were enrolled in the study after giving informed consent. At the initial visit (identified as antenatal visit (ANV) 0), the assistants and the midwives collected information regarding the reproductive and the current pregnancy histories, medical history, socio-economic indicators, and the use of bed nets.

At each monthly ANV, axillary temperature and blood pressure, weight, height and mid-upper arm circumference were measured. After clinical examination, rapid diagnostic tests (RDT) (Parascreen*, Zephyr Biomedical Systems), thick and thin blood smears were systematically made to determine malaria infection. Any time during the follow-up, women were invited to attend health facilities in case of clinical symptoms to get care. The same clinical and biological information were collected as during ANVs. These visits were identified as "unscheduled visits".

On all occasion during follow-up (ANVs, unscheduled visits or delivery), symptoms were actively asked by the midwives in the private examination room following a standardized questionnaire. The symptoms included: headache, fever, stomach ache, nausea or vomiting, fever in the last 48 hours, shivering.

Following the national guidelines, for IPTp two doses of SP (1500 mg sulphadoxine and 75 mg pyrimethamine) were administered at least one month apart during the second trimester of pregnancy under the supervision of midwives during antenatal visits. During the follow-up, a malaria infection was defined as a positive RDT and fever was defined as an axillary temperature above or equal to 37.5°. Following the national guidelines, all participants with malaria parasitaemia as detected by RDT received a treatment dose of quinine (24 mg/kg during 7 days), or SP if they were supposed to receive the scheduled dose on the same visit.

Four ultrasound scans were performed from inclusion until delivery: the first to determine the exact term of the current pregnancy and the following to evaluate intrauterine growth and foetal morphology. At delivery, temperature, weight and blood pressure of the mother were measured. Venous blood samples for biological investigations, thick and thin blood smears and a RDT were obtained before delivery. The newborn child was fully examined.

### Laboratory procedures

Thick blood smears (TBS) were stained with Giemsa and read by two experienced parasitology technicians. Smears were considered negative if no asexual-stage Plasmodium parasite was detected after 500 leucocytes had been counted. Malaria parasites were counted against 200 leukocytes and parasite densities were estimated using leukocyte count of the hemogram. If results were discrepant, the slides were read by a third microscopist.

For statistical analyses, malaria infection was defined as parasitaemia detected by thick blood smear. Geometric means were used to calculate average parasitaemias.

This study was approved by the ethics committees of the Research Institute for Development (IRD) in France and of the Science and Health Faculty (University of Abomey Calavi) in Benin. Written informed consent was obtained from all participants.

### Statistical analysis

Data were double-entered, validated and cleaned using Access (Microsoft, version 2007). Stata version 10 for Windows (Stata Corp, College Station, TX, USA) was used for all statistical analyses. There were two sorts of missing data to deal with: missing symptoms for the first included women and missing malaria status at delivery, essentially because of public health worker's strike. First, the characteristics between the mothers with and without symptoms during first ANVs on one hand and the ones with and without malaria status at delivery on the other hand, were compared to ensure they were similar.

Also, for the women whose symptoms were missing, the multiple imputation by chained equation method (MICE) based on the Gibbs algorithm was used and then the stability of the results was checked with and without MICE. Twenty imputed data sets were carried out with 15 iterations for each.

For missing malaria status, a sensitivity analysis was performed to ensure that the results remained the same, and thus these missing data would not induce a bias. Differences in proportions and means were compared using the khi2 (or Fisher's exact test) and the Student t-test, respectively. A multivariate logistic regression was used to determine the association between signs and symptoms with positive malaria status at each visit. Covariates were included in the initial models only if the p-value was < 0.2 in the univariate analysis. In the final multivariate analysis, a p-value < 0.05 was considered significant.

## Results

**Figure 1:**
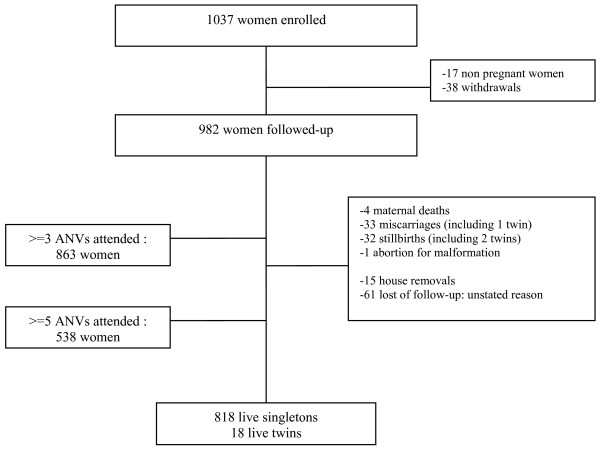
**Study profile**.

Figure 1 shows the flow of women through the study. 1,037 women were enrolled and 55 were excluded (38 withdrawals and 17 non pregnant women). 70 women did not complete their pregnancy for medical reasons (four maternal deaths, 32 stillbirths, 33 miscarriages and one abortion) and 76 were lost to follow-up. The 76 women lost to follow-up had delivered outside the STOPPAM frame and, therefore, outcomes of deliveries were unknown. 836 women were known to have given birth, however 128 TBS had not been performed due to a public health-workers' strike, and malaria status was not available for these women. Nevertheless, age, gravidity, education were similar between women with available TBS at delivery and those without; and between women followed-up and women lost to follow-up.

Table 1 shows the general characteristics of the mothers. On average mothers were 26.4 years old (range 15-45). Among the 982 followed-up women, 179 (18.2%) were primigravidae. At enrolment, the mean gestational age was 17.2 weeks (standard deviation (sd) = 4.7) with a minimum of five weeks. 32.0% of women reported the possession of bed nets at enrolment. Five hundred twenty nine women attended unscheduled visits at least once. The mean numbers of antenatal visits and unscheduled visits were 4.4 (sd = 1.6) and 0.8 (sd = 0.9) respectively and reached a maximum of eight and five visits, respectively.

**Table 1:** General characteristics of the mothers

Factor	Mean (SD) or N (%)
**Age (years)**	26.4 (6.2)
**Gestational age, assessed by ultrasound (weeks)**	17.2(4.7)
**Gravidity**	
1	179 (18.2%)
2	219 (22.3%)
> 2	584 (59.5%)
**Possession of bed net at enrolment**	314 (32.0%)
**Education**	
None	550 (56.0%)
Primary	290 (29.5%)
Secondary	142 (14.5%)
**Number of ANVs**	4.4 (1.6)
**Number of emergency visits**	0.8 (0.9)
**HIV status**	
Negative	846 (86.2%)
Positive	16 (1.6%)
Unknown	120 (12.2%)

The overall prevalence of malaria infections was 10.2% (602/5909). During the whole follow-up, primigravidae had more peripheral infections than multigravidae (15.0% vs 9.2%; p < 0.001). At the initial ANV (prior to treatment and IPTp), the prevalence of malaria infections for primigravidae, secundigravidae and multigravidae were 30.7%, 18.7% and 11.7%, (p < 0,001), respectively.

Table 2 displays the signs and symptoms presented by the mothers at ANVs, unscheduled visits and delivery, and the multivariate analysis of factors associated with a positive TBS at each visit.

**Table 2:** Factors associated with malaria infection (assessed by thick blood smear) during antenatal visits, unscheduled visits and at delivery

		Observed		Univariate analysis	Multivariate analysis after MICE**	
		
		Malaria infected n (%)	No malaria infected n(%)	p-value	Adjusted OR [95% CI]	p-value
**Antenatal visits**						
Fever	No	374 (94.2)	3842 (97.0)	0.003 *		
	Yes	23 (5.8)	118 (3.0)			
Headache	No	239 (71.1)	2907 (81.8)	< 0.001 *	1.9 [1.3-2.6]	< 0.001 *
	Yes	97 (28.9)	648 (18.2)			
Nausea	No	323 (96.1)	3444 (96.9)	0.438		
	Yes	13 (3.9)	110 (3.1)			
IPT	0 dose	256 (64.5)	1434 (36.2)	< 0.001 *	0.6 [0.5-0.7]	< 0.001 *
	1 dose	39 (9.8)	840 (21.2)			
	2 doses	102 (25.7)	1685 (42.6)			
Rainy season	No	207 (52.1)	2150 (54.3)	0.412		
	Yes	190 (47.9)	1810 (45.7)			
Bed net use	No	221 (55.7)	1450 (36.6)	< 0.001 *	1.4 [1.1-1.8]	0.003 *
	Yes	176 (44.3)	2510 (63.4)			
**Unscheduled visits**						
Fever	No	66 (50.8)	616 (88.5)	< 0.001 *	5.2 [3.2-8.1]	< 0.001 *
	Yes	64 (49.2)	80 (11.5)			
Headache	No	24 (18.5)	352 (50.7)	< 0.001 *	2.1 [1.3-3.6]	0.004 *
	Yes	106 (81.5)	342 (49.3)			
Nausea	No	109 (83.9)	605 (87.1)	0.326		
	Yes	21 (16.1)	90 (12.9)			
Feverishness in the last 48 hrs	No	54 (41.5)	498 (72.6)	< 0.001 *		
	Yes	76 (58.5)	188 (27.4)			
Shivering	No	59 (45.4)	567 (81.6)	< 0.001 *	3.11 [2.0-4.9]	< 0.001 *
	Yes	71 (54.6)	128 (18.4)			
IPT	0 dose	33 (25.4)	140 (20.1)	0.058		
	1 dose	15 (11.5)	138 (19.8)			
	2 doses	82 (63.1)	418 (60.1)			
Rainy season	No	70 (53.9)	414 (59.5)	0.231		
	Yes	60 (46.1)	282 (40.5)			
Bed net use	No	33 (25.4)	193 (27.7)	0.582		
	Yes	97 (74.6)	503 (72.3)			
**Delivery**						
Primigravidae	No	54 (76.1)	537 (84.3)	0.076		
	Yes	17 (23.9)	100 (15.7)			
Age under 25	No	38 (53.5)	384 (61.4)	0.196		
	Yes	33 (46.5)	241 (38.6)			
Fever	No	59 (83.1)	601 (94.4)	< 0.001 *	3.6 [1.7-7.7]	0.001 *
	Yes	12 (16.9)	36 (5.7)			
Headache	No	63 (92.7)	610 (98.2)	0.004 *	3.6 [1.1-11.6]	0.029 *
	Yes	5 (7.3)	11 (1.8)			
Nausea	No	66 (97.1)	609 (98.1)	0.576		
	Yes	2 (2.9)	12 (1.9)			
Rainy season	No	38 (53.5)	360 (56.5)	0.630		
	Yes	33 (46.5)	277 (43.5)			
Bednet use	No	13 (18.3)	113 (17.7)	0.905		
	Yes	58 (81.7)	524 (82.3)			

* Statistically significant results (p < 0.05)

** adjusted on women's characteristics (parity and age)

As the STOPPAM project was not originally designed to assess malaria clinical presentation during pregnancy, the first 126 included women were not asked about symptoms. Symptoms during first ANVs were thus lacking for these women. To deal with these missing data, the MICE method based on the Gibbs algorithm was used.

During ANVs (including enrolment), among the 982 women initially followed-up, 307 were infected at least once. Women under 25 (39.6% vs 25.6%; p < 0.001) and primigravidae (43.0% vs 28.6%; p < 0.001) had a higher risk of peripheral malaria infections than older women and multigravidae, respectively.

On a total of 4,357 visits, 397 malaria infections were found (table 2). Considering the total number of ANV, the only clinical symtpom significantly associated with a higher risk of positive TBS was headache (aOR = 1.9; p < 0.001). Fever was also associated with malaria infection in univariate analysis (p = 0.003), but this association was not significant anymore in multivariate analysis (aOR = 1.6; p = 0.069). The intake of SP was protective (aOR = 0.6; p < 0.001) and to not use bed nets was at higher risk of malaria infection (aOR = 1.4; p = 0.003). Among the 397 malaria infections occurring during ANVs, headache was reported in 97 cases.

Among the 524 women who consulted during unscheduled visits, 114 were infected at least once. Young age of the woman was a risk factor to develop a parasitaemia (26.4% vs 19.2%; p = 0.05).

On a total of 826 unscheduled visits, 130 malaria infections were found (table 2): 99 women were infected once, 14 women twice and one woman thrice. Considering the total number of unscheduled visits, fever (aOR = 5.2; p < 0.001), headache (aOR = 2.1; p = 0.004), and shivering (aOR = 3.1; p < 0.001) were significantly associated with a malaria infection during unscheduled visits. One hundred and sixteen of the 130 malaria episodes occurred with one or more of these symptoms, which represents 89% of the malaria infections during unscheduled visits.

Analyses were performed with and without MICE method and the results remained unchanged.

At delivery, 71 women were infected with malaria and fever and headache were significantly more frequent in positive TBS mothers (aOR =3.6; p =0.001 and aOR =3.6; p =0.029 respectively, table 2). A sensitivity analysis was performed to ensure that the results remained the same, and thus these missing data would not induce a bias.

In addition, considering any symptom associated to malaria infection, the mean parasitaemia of symptomatic malaria infections was much higher during unscheduled visits than during ANVs (3641 parasites/μL vs 420 parasites/μL; p < 0.001).

**Figure 2:**
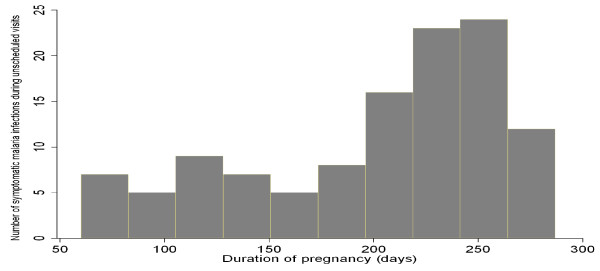
**Distribution of symptomatic malaria infections during unscheduled visits**.

Based on the pharmacokinetic properties of SP,[14] we analysed the occurrence of malaria infections during unscheduled visits in relation to a presumed SP efficacy period of two months for each subject. 67% of malaria infections occurred when the last dose of SP had been given at least two months before (67% vs 33%, p < 0.001). As shown in Figure 2, two thirds of the symptomatic malaria infections occurred in late pregnancy, after the 6^th ^month.

## Discussion

According to what has been generally described in stable endemic areas, the majority (60%) of pregnant women who were followed-up in the STOPPAM project were asymptomatic when infected, although some of them displayed clinical signs. During ANVs, 5% of the infected women were febrile; but only headache, a non-specific symptom, was significantly associated with a higher risk of having a peripheral infection. However, its frequency was moderate (28.9% of the malaria infections). In contrast, during unscheduled visits, Almost 90% of the malaria episodes were symptomatic and fever (nearly half of the parasitized women), headache and shivering were strongly associated with malaria parasitaemia.

In high transmission areas pregnant women still have an immune protection, and only exhibit limited symptoms when they get infected by malaria parasites[6-8]. Therefore, only a limited number of studies focused on these issues and their results were often inconclusive. A cross-sectional study conducted in Mozambique, limited pregnant women consulting with clinical complaints found that three quarters of them exhibited symptoms suggestive of malaria, although only one quarter of them were parasitaemic [10]. Another study in Nigeria also found a very low specificity of fever in the prediction of malaria infection in pregnant women [11].

A third study carried out in Ghana reported that MiP was often symptomatic when women were actively asked for clinical complaints[12]. All women attending the antenatal clinics were screened for malaria without distinction between scheduled or unscheduled visits. Malaria infected women were enrolled into a randomized controlled trial and women showing no malaria parasitaemia were selected as a control group. However, because of the case-control design of this study, and the lack of blinding on the disease (presence of parasites in peripheral blood), there may have been a risk of over-reporting of these symptoms.

The STOPPAM project was able to offer women both prospective routine ANVs and unscheduled visits in case of clinical complaints. Also, symptoms and signs were actively asked by midwives before TBS were performed. Therefore, it was not prone to selection biases unlike the abovementioned studies. Our results show that the majority of infected pregnant women in this stable malaria area only have few and mild clinical signs during routine visits. Unscheduled visits are frequently motivated by malaria-associated acute symptoms, particularly at distance from IPTp intakes. These women are important to identify and to treat, in order to avoid adverse consequences of MiP on the newborn child.

In the statistical analysis, missing data related to symptoms for the first women included were an issue to deal with. First, gestity, age and education were shown not to be different between the women with missing data and the remaining women. Then, the MICE method for these missing data was used and all results remained similar using the MICE method or not. Therefore, missing data related to symptoms were unlikely to have induced a bias, and then to have altered the results.

For the delivery, malaria status was missing for 140 women who were thus excluded from the analysis. Characteristics between excluded and remaining women were similar and the sensitivity analysis performed ensured that the results were similar. Moreover, the reason for excluding these women (health worker's strike) could not possibly influence malaria status at delivery. Consequently, the mechanism of missing data could not have altered the results and induced a selection bias.

According to the few pharmacokinetic studies available, the SP blood concentration effective against a sensitive *Plasmodium *isolate appears not to last over two months in non-pregnant women [9,14], hence this duration was chosen as an estimate of the efficacy of SP. In pregnant women, the pharmacokinetics of drugs are modified due to an expanded volume of distribution, and the duration of efficacy may actually be shorter. In Benin, in vivo resistance rates to SP up to 50% have been evidenced in children in a neighbouring area to our study [15]. Even considering a hypothetical long duration of efficacy, more than two thirds of malaria infections we observed in this study occurred on the occasion of unscheduled visits when SP could not be considered efficacious any more, mostly at the end of the pregnancy. As the foetal growth is more important during the last trimester, these infections could be the most deleterious for the foetus.

To avoid such late infections, different options could be considered to offer pregnant women a better protection. In sub-Saharan Africa, the attendance to ANVs is rather high: almost 70% of women attend at least once, 95% of them attend twice and more than half attend four times[1,16]. The WHO recommends four ANVs during pregnancy including three after quickening[1]. To improve the IPTp strategy, a possibility would be to space out the interval between two SP doses. A first dose could be given at the first ANV after quickening and a second dose at least eight to 10 weeks later, instead of the recommended space of one month.

Another option to improve the coverage of IPTp is to give a third dose of SP. This strategy is currently recommended for HIV-positive pregnant women, but few studies have evaluated the benefits of increasing the number of SP administrations in HIV-negative women[17-19]. Besides, safety information on the consequences of increasing number of SP doses are lacking, as it could also widespread resistance to the drug. It has been documented that the administration of SP through IPTi to infants in high transmission areas has little impact on the resistance[20,21]. One can assume that the use of SP in pregnant women, which is a smaller population than infants, would have minor consequences. A clinical trial is currently ongoing in Mali (A. Dicko, personal communication) and thus should bring valuable information on the subject.

## Conclusion

This study found that pregnant women infected with Plasmodium only had symptoms related to malaria when infection was detected at unscheduled visits, generally at distance from the last dose of IPTp. A third dose of IPTp administered at the end of pregnancy might avoid such late infections. An alternative to SP-IPTp in endemic areas where malaria incidence is declining or SP resistance is high, could be an "intermittent screening and treatment", especially in areas with high seasonal transmission, which consists in screening all pregnant women during ANVs with RDTs and to treat only malaria infected women.

## Competing interests

The authors declare that they have no competing interests.

## Authors' contributions

BTH carried out the acquisition, the statistical analysis and the interpretation of data and drafted the manuscript. NF participated in the design and the coordination of the study, the acquisition of data and revised critically the manuscript. GB and SB participated in the acquisition of data. BGM and AM participated in the design of the study and revised critically the manuscript. PD coordinates the STOPPAM project, he participated in the design of this study and revised critically the manuscript. MC participated in the design and the coordination of the study and supervised the writing of the manuscript. All authors read and approved the final manuscript.
